# Familial breast cancer: Genetic counseling over time, including patients´ expectations and initiators considering the Angelina Jolie effect

**DOI:** 10.1371/journal.pone.0177893

**Published:** 2017-05-25

**Authors:** Christina Evers, Christine Fischer, Nicola Dikow, Sarah Schott

**Affiliations:** 1 Institute of Human Genetics, Heidelberg University, Heidelberg, Germany; 2 Department of Gynecology and Obstetrics, University Hospital Heidelberg, Heidelberg, Germany; 3 German Consortium for Translational Cancer Research (DKTK), Heidelberg, Germany; Ohio State University Wexner Medical Center, UNITED STATES

## Abstract

**Purpose:**

The German Consortium for hereditary breast/ovarian cancer (GC-HBOC) aims for nationwide access to professional, individualized yet structured care for families at high risk. The identification of such families remains key for optimal care. Our study evaluates counselees’ characteristics, referral practices, expectations and motivations in respect to their first genetic consultation. The impact of the Angelina Jolie Effect (AJE) was prospectively assessed.

**Methods:**

All counselees could participate through a questionnaire. Groups were built in respect to neoadjuvant chemotherapy (FT) and before/after AJE.

**Results:**

The 917 (88.5%) counselees (FT: 8.2%) were on average female (97.3%), with a mean age of 44.6, had children (71.9%), higher education (88%), personal (46.4%) or at least one first-degree relative (74.6%) with BC/OC or known *BRCA1*/2 mutation (11.8%), were in a relationship (76.1%), and living in a village (40.7%). The AJE is associated with significantly fewer cancelations (p = 0.005), more attendance among men (4.2% vs. 0.8%, p = 0.002), and people with familial *BRCA1/2* (14.8% vs. 7.5%, p = 0.003). The majority seek information regarding their cancer risk (83%) or relatives’ risk (74.8%), HBOC (69.1%), and surveillance programs for themselves (66.6%) or relatives (60.6%).

**Conclusion:**

Enhanced media awareness of genetic cancer motivates patients, including other patient groups. A higher number of participants, including more men, are attending GC due to the AJE. In terms of the rising complexity of genetic testing, the analysis of patients’ expectations and initiators for GC suggests that there is an urgent need to develop to participate motivation analysis. The factors revealed as impediments to accessing GC-HBOC guide recommendations to optimize access to genetic counseling. Medical educational programs for primary gynecologists and families at risk might be options to reach more participants.

## Introduction

Breast cancer (BC) is the leading cancer among women worldwide and is of hereditary origin in 5–10% of cases, mainly due to BRCA1/2 mutations [1]. Mutations in other genes currently play a minor role because of their low prevalence or reduced penetrance. Female BRCA mutation carriers face an elevated lifetime cancer risk of up to 60% for BC and ovarian cancer (OC) [2] and an age-dependent contralateral BC risk of up to 55% [3]. Since the discovery of the BRCA mutation in the ´90s, several health care programs have been established worldwide, such as the German Consortium for hereditary BC/OC with its 17 specialized academic centers (http://www.konsortium-familiaerer-brustkrebs.de/). Its aim is nationwide access to professional, individualized structured care for families at high risk for HBOC. This includes interdisciplinary genetic, gynecological, and psychosomatic counseling with an individual risk assessment on the basis of a detailed pedigree and molecular genetic testing for those who fulfill the criteria proposed by GC-HBOC [4]. An intensified BC surveillance program is recommended depending on patients’ age, morbidity, and personal preferences, even when individuals test negative for *BRCA1* or *2* mutations if the family history indicates a “high risk” for BC through pedigree analysis [5]. This surveillance is well accepted and includes regular breast MRI, mammography, ultrasound, and a physical exam, all financed by the German health insurances [6]. Prophylactic surgery is also a topic of discussion for BRCA mutation carriers.

The identification of women and families at high risk remains key to offering optimal care strategies; however, only half of patients identified as high risk for BC are referred for BRCA counseling in the US, indicating an excessive under use of this important type of health care, especially among minorities [7]. Prior studies have shown that this lack of access is correlated with socioeconomic factors and lack of awareness of this type of testing among minorities [8–12]. Healthcare provider referrals seem to have the strongest influence on attending genetic counseling [12]. However, experiences from other countries show that 14–27% of women with HBOC seek genetic counseling without recommendation from medical professionals; data for Germany have not yet been evaluated [13, 14].

In May 2013, Angelina Jolie (AJ), brought huge public attention to HBCO by going public with her choice to undergo prophylactic mastectomy due to her BRCA mutation. The “Angelina Jolie Effect” (AJE) is now described in literature as having caused a major impact on genetic enquiries [15, 16]. Our study was conducted to evaluate the counselees’ general characteristics, how they arrived at the decision to undergo genetic counseling, and their expectations and motivations in respect to their first visit. The impact of the AJE has been prospectively assessed, and the factors revealed as impairments to accessing HBOC centers have guided recommendations to optimize genetic HBOC counseling within Germany.

## Material and methods

### Study design, participants and data collection

All counselees attending their first appointment between September 2012 and January 2015 at the interdisciplinary Center for HBOC at the University Hospital in Heidelberg, Germany, were eligible to participate by filling out a questionnaire. In Germany, families with a probability of mutation >10%, meaning they either have a known BRCA mutation or a familial accumulation of HBOC cases, are eligible to undergo genetic testing at one of the HBOC centers [4]. These expenses are then covered by public insurance. Prior to the initial appointment, a telephone interview is performed to ask about family cancer cases, which is important for the insurance coverage criteria. If applicable, the families are then invited to an interdisciplinary consultation appointment; otherwise they are not scheduled for an appointment. The first visit includes a visit with a medical doctor from the genetics department as well as a gynecologist. During this first visit, a pedigree is drawn and a risk calculation using Cyrillic 2.1. software is performed (www.cyrillicsoftware.com) by a medical doctor specialized in genetics. Afterwards, the surveillance strategies or options for prophylactic surgery are discussed with a gynecologist. However, the person with a personal history of cancer within the family needs to be tested first by law. If a mutation is detected, all other family members are permitted to undergo testing. If the family members with a cancer history are deceased, healthy individuals are also allowed by law to have genetic testing.

Participation in the study was possible if the following criteria were met: a) an indication for genetic counseling as outlined by the German HBOC, confirmed on the phone prior to their visit, b) sufficient intellect and language skills to complete the questionnaire, and c) written informed consent was provided. The study sheets were handed out by the study nurse, and participants were checked for inclusion criteria upon arrival. This study was performed in accordance with the Declaration of Helsinki after approval by the local ethics committee (Ethikkommission der Medizinischen Fakultät Heidelberg, Heidelberg Germany, number S-303/2012). Counselees without a known familial *BRCA1/2* mutation were defined as “high risk” if their mutation carrier probability was >20% or their remaining lifetime risk for breast cancer was >30% as calculated using the pedigree software Cyrillic 2.1. A subset of counselees with neoadjuvant chemotherapy were prioritized as “fast track” (FT). For these patients, an appointment for counseling was arranged as quickly as possible (usually within 4 weeks), and their samples were processed first. Most FT women decide to undergo contralateral prophylactic mastectomy or mastectomy versus breast conserving treatment. Therefore these results are needed prior to surgery in order to support informed consent decision making. All remaining counselees were defined as “non-fast track” (NFT). There was no urgent surgical treatment decision based upon genetic test results for this group. PARP (Poly(ADP-ribose)-Polymerase)) treatment was not approved in Germany at the time of the observational period; for women who were treated in PARP-studies, somatic testing was performed. For statistical analysis, FT patients were evaluated separately.

The study started prior to the AJE, and participants were analyzed in terms of its impact on counselees´ characteristics, referral patterns and motivational factors. The “before AJ” group comprises all study participants included on or before the 31^th^ of August 2013; the group’s median waiting time for an appointment was around three months as of May 2013. The “after AJ” group comprises all participants included from October 2013 onwards. All counselees contacting the center were screened for inclusion criteria as stated above. There were many families that did not meet the criteria for further testing and never got scheduled. Therefore, the total inquiries were not countable but all people who attended had to fulfilled the criteria for HBOC consultation.

### Questionnaire

A specially designed questionnaire consisting of 34 items was designed by the authors based on the experiences of previous studies (S1 File). The questions comprised the following main issues and could be answered within 10 minutes:

1. Counselee characteristics: socio-demographic aspects, such as age, gender, number of children, marital status, country of origin, hometown (countryside, town or city), education, and employment status. Medical and familial histories were recorded, including personal history of BC and/or OC, personal “high risk” situation for BC, presence of at least one first-degree relative with BC and/or OC and/or presence of a familial *BRCA1/2* germline mutation.
2. Access to the Center: Transportation to the center, the waiting time for an appointment, and the time interval between first learning about the opportunity for genetic counselling and actually initiating an appointment by the counselee were recorded. In addition, prior appointment cancelations by counselees were asked about.
3. Initiator of the referral: The main players in the initiation of counseling and referral patterns were evaluated. In order to improve outreach and awareness, knowledge about these aspects was necessary.
4. Expectations and motivations of the counselees: Counselees´ expectations and motivation regarding their first consultation were questioned.

### Motivational groups

For further data analysis, the NFT counselees were divided into three motivational groups. Motivation was defined if a ≥3 was marked. Group M1 includes all counselees interested (scale ≥3) in obtaining information about their own cancer risk, their own surveillance, and the cause of their cancer, addressing 7 motivational factors. Group M2 comprises counselees that stated interest in their own cancer risk and surveillance. M3 includes all counselees interested in at least the cause of their cancer. Motivation groups were overlapping, meaning that one patient could appear in more than one group.

### Statistical analysis

This was an observational study, and the statistical analyses were performed by a statistician (CF) using SPSS 21 (IBM Corp. Armonk, NY, USA). Continuous data were reported as means with standard deviations and categorical data as absolute and relative frequencies. For description analysis T-tests were used to test for differences between groups in the case of continuous data, while the Chi-squared test or exact Fisher test evaluated differences for categorical data. P-values <0.05 (two-sided) were regarded as statistically significant and were not corrected for multiple testing.

## Results

### Study population

**Fig 1:**
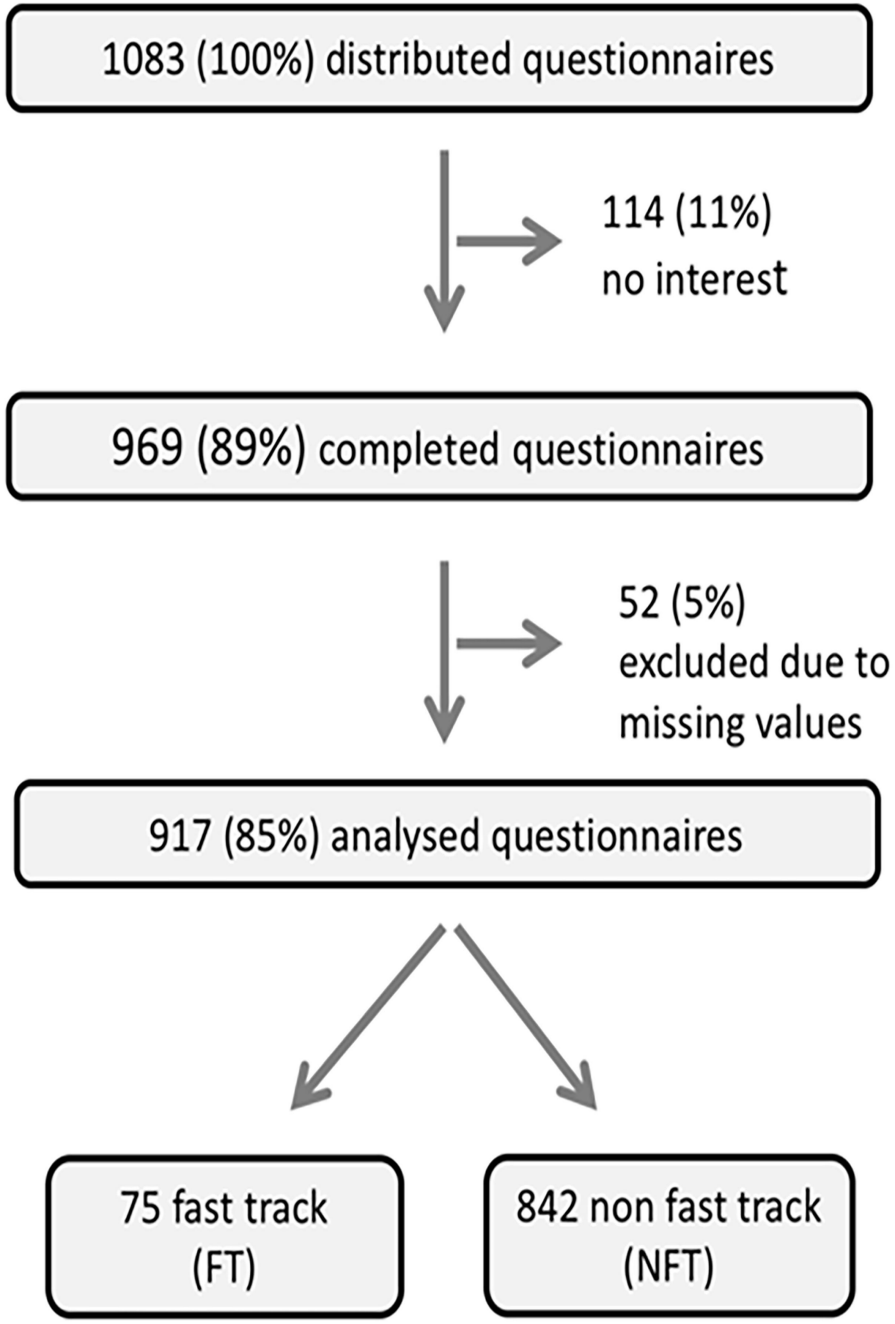
Study participants.

One thousand eighty-three counselees received the questionnaire. 969 (89%) returned the questionnaire; 114 (11%) declined to participate. 52 (0.5%) were excluded due to missing values. In total, 917 (88.5%) counselees formed the study population (Fig 1). Informed consent was obtained from all participants individually. 75 patients (8.2%) were categorized as FT patients and were considered separately. Detailed data on all NFT (n = 842) and FT (n = 75) counselees are given in Table 1. The 842 NFT counselees’ (91.2%) characteristics are shown in Table 1. In brief, the NFT group comprised 819 (97.3%) women and 23 men (2.7%), resulting in a male to female ration of 1:39. The counselees had a mean age of 44.6 years (SD 12.54). Most NFT participants had children (71.9%), were married or in a relationship (76.1%), and lived in a village (40.7%) or small town (39.6%). The vast majority were German (89.2%), and 88% had a higher level of education (finished high school, college, professional training or university). The median travel distance to our Center was 80.3 km, but this ranged widely (+/-66.76 km). Seven percent (n = 58) had cancelled at least one appointment prior to their first attended visit. Nearly half of the NFT patients (46.4%) had a personal history of BC/OC, 74.6% had at least one first-degree relative with BC/OC, and 11.8% had a known familial *BRCA1* or *BRCA2* mutation. Of the 742 counselees without a known *BRCA1/2* mutation, 476 (64.2%) were in a high risk situation by pedigree.

**Table 1:** Socio-demographic data of the entire study population (NFT and FT). Characteristics of all counselees and differences between the NFT and FT groups.

	Study population		NFT		FT		p-value
**Gender** female/male (% fem)	894/23	(97.5%)	819/23	(97.3%)	75	-100%	0.137*
**Age**, years	44.2	±12.43	44.6	± 12.54	39.2	± 9.87	< 0.001
**Distance**, km	79.0	± 65.79	80.3	± 66.76	65.2	± 52.07	0.058
**Children** y/n (%y)	645/264	(71.0%)	600/234	(71.9%)	45/30	(60.0%)	0.022
**Marital status**							
single	128	(14.0%)	116	(13.9%)	12	(16.0%)	0.008*
married	574	(62.9%)	537	(64.2%)	37	(49.3%)	
in a relationship	118	(12.9%)	100	(11.9%)	18	(24.0%)	
divorced	65	(7.1%)	57	(6.8%)	8	(10.7%)	
widowed	27	(3.0%)	27	(3.2%)	0	(0.0%)	
**Origin**							
Germany	812	(89.2%)	747	(89.4%)	65	(87.8%)	0.690
other	98	(10.8%)	89	(10.6%)	9	(12.2%)	
**Hometown**							
village	367	(40.5%)	338	(40.7%)	29	(38.7%)	
small town	355	(39.2%)	329	(39.6%)	26	(34.7%)	
city	184	(20.3%)	164	(19.7)	20	(26.7%)	
**Education**							
none	3	(0.4%)	3	(0.4%)	0		0.840*
lower school	95	(11.1%)	89	(11.3%)	6	(8.7%)	
high school	83	(9.7%)	77	(9.8%)	6	(8.7%)	
college	135	(15.8%)	125	(15.9%)	10	(14.5%)	
profession	314	(36.6%)	290	(36.8%)	24	(34.8%)	
university degree	227	(26.5%)	204	(25.9%)	23	(33.3%)	
**Profession**							
in training	45	(5.0%)	42	(5.1%)	3	(4.2%)	0.026*
employee	588	(65.3%)	531	(64.1%)	57	(79.2%)	
freelancer	54	(6.0%)	52	(6.3%)	2	(2.8%)	
housewife	104	(11.5%)	98	(11.8%)	6	(8.3%)	
pensioner	88	(9.8%)	87	(10.5%)	1	(1.4%)	
none employed	22	(2.4%)	19	(2.3%)	3	(4.2%)	
**Breast or Ovarian Cancer History**							
yes /no (yes%)	465/451	(50.8%)	390/451	(46.4%)	75/0	-100%	< 0.001
**High risk situation**							
yes / no (yes%)	518/298	(63.5%)	476/266	(64.2%)	42/31	(56.8%)	0.129
**1st degree relative with BC/OC**							
yes / no (yes%)	651/261	(71.4%)	625/213	(76.6%)	26/48	(35.1%)	< 0.001
**BRCA mutation within the family**							
yes / no	108/807	(11.1%)	108/733	(12.8%)	0/74	(0.0%)	< 0.001*
**First appointment cancelled**							
yes / no	62/849	(68.1%)	59/778	(7.0%)	4/71	(5.3%)	0.109

p-values refer to two-sided tests, t-tests for quantitative variables, * Fisher's exact test, Chi2 tests otherwise quantitative variables are given as mean ± standard deviation; based on 75 fast track (FT) and 842 non fast track persons (NFT)

The subset of patients with FT prioritization were significantly younger (39.2 vs. 44.6 years, p = 0.001), had first-degree relatives with BC/OC less frequently (35.1% vs. 76.6%, p = 0.001), and were in a high risk situation by pedigree less frequently (56.8% vs. 64.2%, p = 0.129). None of the FT patients had a known familial *BRCA1*/2 mutation.

The NFT counselee data were analyzed in respect to the Angelina Jolie Effect (AJE) (Table 2). In the NFT group, the waiting time greatly increased by an average of 50 days after Angelina Jolie’s public disclosure regarding her *BRCA* status. Significantly fewer appointments were cancelled (4.7% vs. 10.0%, p = 0.005), significantly more men (4.2% vs. 0.8%, p = 0.002) and counselees with a known *BRCA1/2* mutation in the family (14.8% vs. 7.5%, p = 0.003) attended the HBOC consultation. In addition, counselees tended to be older (45.4 vs. 43.7 years) and more often have children (74.9% vs. 68.1%).

**Table 2:** Characteristics of NFT counselees and influence of the “Angelina Jolie (AJ) effect” on them (“before AJ” and “after AJ” subgroups).

	NFT study population		before AJ		after AJ		p-value
**Gender** female/male (% female)	819/23	(97.3%)	365/3	(99.2%)	454/20	(95.8%)	0.002
**Age**, years	44.6	±12.54	43.7	± 12.41	45.4	± 12.60	0.047
**Distance**, km	80.3	± 66.76	76.7	± 54.52	83.0	± 74.61	0.178
**Children** yes/no (%yes)	600/234	(71.9%)	248/116	(68.1%)	352/118	(74.9%)	0.036
**Marital status**							
single	116	(13.9%)	55	(15.9%)	61	(12.9%)	0.672*
married	537	(64.2%)	228	(62.5%)	309	(65.5%)	
in a relationship	100	(11.9%)	48	(13.2%)	52	(11.0%)	
divorced	57	(6.8%)	22	(6.0%)	35	(7.4%)	
widowed	27	(3.2%)	12	(3.3%)	15	(3.2%)	
**Origin**							
Germany	228	(89.2%)	65	(87.8%)	747	(89.4%)	0.219
other	98	(10.8%)	9	(12.2%)	89	(10.6%)	
**Hometown**							
village	338	(40.7%)	152	(41.9%)	185	(39.7%)	0.237
small town	329	(39.6%)	149	(41.0%)	180	(38.5%)	
city	164	(19.7%)	62	(17.1%)	102	(21.8%)	
**Education**							
none	3	(0.4%)	0		3	(0.7%)	0.005
lower school	89	(11.3%)	52	(15.0%)	37	(8.4%)	
high school	77	(9.8%)	37	(10.7%)	40	(9.1%)	
college	125	(15.9%)	61	(17.6%)	64	(14.5%)	
profession	290	(36.8%)	109	(31.4%)	181	(41.0%)	
university degree	204	(25.9%)	88	(25.4%)	116	(26.3%)	
**Profession**							
in training	42	(5.1%)	22	(6.1%)	20	(4.3%)	0.725
employee	531	(64.1%)	236	(65.2%)	295	(63.2%)	
freelancer	52	(6.3%)	19	(5.2%)	33	(7.1%)	
housewife	98	(11.8%)	41	(11.3%)	57	(12.2%)	
pensioner	87	(10.5%)	36	(9.9%)	51	(10.9%)	
none employed	19	(2.3%)	8	(2.2%)	11	(2.4%)	
**BC/OC history**							
yes /no (yes%)	390/451	(46.4%)	174/193	47.4%	216/258	(45.6%)	0.595
**High risk situation**							
yes / no (yes%)	476/266	(64.2%)	111/121	(64.7%)	254/145	(63.7%)	0.763
**1st degree relative with BC/OC**							
yes / no (yes%)	625/213	(74.6%)	267/99	(75.0%)	358/114	(75.8%)	0.339
**BRCA mutation within the family**							
yes / no (yes%)	99/742	(11.8%)	29/359	(7.5%)	70/403	(14.8%)	0.003
**Appointment cancelled**							
yes / no (yes%)	58/778	(7.0%)	36/329	(10.0%)	22/449	(4.7%)	0.005

p-values refer to two-sided tests, t-tests for quantitative variables, Chi^2^ tests otherwise, * Fisher's exact test, quantitative variables are given as mean ± standard deviation, based on 842 non fast track persons (NFT)

### Referral patterns

The referral patterns describe the initiators and referrer to our HBOC Center. Detailed information regarding all counselees and the NFT/FT subgroups are given in S1 Table. The most frequently mentioned initiator of referral among NFT was the primary gynecologist, who was involved in 29.5% of referrals, followed by family/friends (18%), self-referral (13.7%), medical professionals (MP) from external hospitals (12.8%), and Heidelberg University Hospital (11.9%) (multiple answers possible). In contrast, the FT counselees were predominantly referred by our institution (41.9%) and external hospitals (24.8%) followed by the primary gynecologist (12.4%). Referral by family/friends and self-referral in the FT subgroup were rare (5.7% and 4.8%, respectively).

In order to evaluate the number of referrals initiated by an MP in respect to the AJE, we formed three groups: a) counselees referred exclusively by an MP (group 1), b) counselees for which the MP was one of the initiators, and c) counselees coming exclusively upon recommendation by family/friends (Table 3a).

**Table 3:** Initiator groups for genetic counseling. Referral by **a)** medical professionals/physicians and **b)** Heidelberg University Hospital in the NFT and FT groups and the NFT subgroups “before AJ” and “after AJ”.

**a**	**Initiator group**	**NFT**		**FT**		**p-value**	**NFT** **before AJ**		**NFT** **after AJ**		**p-value**
		N	%	N	%		N	%	N	%	
	**1** (only physicians)	433	51.6	60	80.0	<0.0001	213	58.0	220	46.6	0.004
	**2** (both)	189	22.5	10	13.3		69	18.8	120	25.4	
	**3** (without physicians)	217	25.9	5	6.7		85	23.2	132	28.0	
	sum	839		75			367		472		
**b**	**Initiator**	**NFT**		**FT**		**p-value**	**NFT** **before AJ**		**NFT** **after AJ**		
		N	%	N	%		N	%	N	%	
	university hospital	141	16.8	44	58.7	<0.0001	65	17.8	76	16.1	0.51
	other	696	83.2	31	41.3		300	82.2	396	83.9	
	sum	837		75			365		472		

Multiple entries permitted, based on 75 fast track (FT) patients and 837 non-fast track individuals (NFT)

As expected, referral patterns of FT and NFT counselees were significantly different. Counselees in the FT group were more often exclusively referred by an MP compared to the NFT counselees (80% vs. 51.6%, p < 0.0001, Table 3a). Only 6.7% of the FT counselees stated that they came without referral from an MP, while 25.9% in the NFT group came exclusively on the initiative of family/friends (Table 3a). In addition, there was a highly significant (p < 0.001) difference between NFT and FT regarding referrals by our hospital: in the NFT group, only 16.8% came upon our referral; 83.2% had other referral sources. In the FT group, the majority (58.7%) were referred by our MP (Table 3b). For further data analysis, the NFT was sub-grouped regarding age (<55 and ≥55 years), number of children, educational status (higher level of education defined as finished high school, college, professional training or university), history of BC/OC, high risk situation, first-degree relative with BC/OC, familial BRCA mutation, and referral before and after AJ´s decision to go public. Women <55 years of age, those with a higher level of education, a history of BC/OC, and familial BRCA mutation were significantly (p< 0.05) more often referred by an MP (data not shown). We detected an AJE on referral patterns (see also S1 Table). After Angelina Jolie went public with her decision, the number of NFT counselees exclusively referred by an MP decreased from 58.0 to 46.6% (p = 0.004, Table 3a). In the “after AJ” group, slightly more counselees came exclusively upon recommendation by family/friends, although the effect was not significant (23.2% vs. 28%). There was no significant influence of the AJE on the proportion of NFT counselees referred by our hospital (17.8% “before AJ” vs. 16.1% “after AJ”, Table 3b).

### Expectations and motivations of the counselees

Table 4 shows the NFT counselees’ main reasons for attending. The vast majority had a strong interest (score 4 applies fully) in information about their own cancer (83%) and their relatives’ risks (74.8%). Other main motivational factors were general HBOC information (69.1%) and surveillance programs for themselves (66.6%) and relatives (60.6%). The question regarding information about causes of cancer in their own case divided the counselees; 42.5%, were not strongly interested (scale 0), and 31.4% were strongly interested (scale 4) in the etiology of their cancer. Only 14.4% had a strong interest in information about inheritance of HBOC that might be relevant for their family planning. 5.1% had no clear expectations, and 14.6% visited the HBOC Center only upon the recommendation of their physician, relatives, or other initiators (Table 4). Women with BC or OC had significant differences for most of the expectations (p<0.001). They had less interest in their own cancer risk (80.8% vs 92.0%, scale 4), expected information on the cancer risk of their relatives more often (86.6% vs 68.6%, scale 4), fewer of them were interested in early cancer detection surveillance or aftercare programs (55.7% vs 80.5%, scale 4), but more wanted to be informed about early detection or surveillance strategies for family members (75.2% vs 51.3%, scale 4), fewer of them were interested in information concerning their own children (12.6% vs 17.9%, scale 4), and considerably more were interested in knowing the cause of their breast cancer (60.5% vs 9.8%, scale 4).

**Table 4:** Expectations of NFT counselees. Counselees quantified different expectations and motivational factors for visiting the Center for HBOC using a scale that ranged from 0 (motivation factor does not apply) to 4 (motivation factor applies completely).

	0		1		2		3		4		missing	
	N	%	N	%	N	%	N	%	N	%	N	%
**General information (HBOC)**	31	3.7	31	3.7	78	9.3	90	10.7	582	69.1	30	3.6
**Own cancer risk**	32	3.8	15	1.8	23	2.7	34	4.0	699	83.0	39	4.6
**Cancer risk for relatives**	46	5.5	19	2.3	56	6.7	67	8.0	630	74.8	24	2.9
**Own surveillance**	67	8.0	39	4.6	63	7.5	78	9.3	561	66.6	34	4.0
**Surveillance for relatives**	86	10.2	33	3.9	90	10.7	98	11.6	510	60.6	25	3.0
**Cancer aetiology**	361	42.9	33	3.9	70	8.3	39	4.6	264	31.4	75	8.9
**Family planning**	548	65.1	29	3.4	35	4.2	45	5.3	121	14.4	64	7.6
**No personal expectations**	468	55.6	80	9.5	116	13.8	78	9.3	43	5.1	57	6.8
**Visit on recommendation**	492	58.4	61	7.2	82	9.7	49	5.8	123	14.6	35	4.2
**Other expectations than above**	664	78.9	23	2.7	15	1.8	6	0.7	6	0.7	128	15.2

scale 0–4 from "completely no" to "completely yes", row percentages; based on 842 subjects, multiple answers possible

The expectations of the FT subgroup are similar to those of the NFT, including high interest in their own cancer risk (84.0%) and that of their family (78.7%) as well as surveillance programs for themselves (52.0%) and their relatives (56.0%) (S2 Table). A notable difference is the much higher interest (scale = 4) in the cause of their cancer among FT patients (57.3% vs. 31.4%, Table 4, S2 Table).

For further data analysis, the NFT counselees were divided into three motivational groups as stated above. 23.6% of counselees were in group M1, 53.7% M2, and 36.0% M3 (Table 5). The comparison of NFT counselees “before AJ” and “after AJ” showed no significant difference regarding the distribution of the counselees among these three groups (Table 5). Compared to all NFT, the NFT counselees in M1 suffered significantly more often from BC/OC themselves (p < 0.0001) and had a first-degree relative with BC and/or OC significantly more often (p < 0.0001). The M2 counselees were also significantly more often affected by BC and/or OC themselves (p = 0.003). In addition, being in M2 positively correlates with having children (p < 0.0001). Being M3 positively correlates with a medical history of BC and/or OC (p < 0.0001), having at least one first-degree relative with BC and/or OC (p < 0.0001), having children (p < 0.0001), a higher education (p < 0.0001), and being of younger age (<55 years, p < 0.0001) (data not shown).

**Table 5:** Motivation groups for NFT counselees before and after AJ.

	All NFT		NFT before AJ		NFT after AJ		
	N	%	N	%	N	%	p
**M 1**							
no	643	76.4	282	76.6	361	76.2	0.470
yes	199	23.6	86	23.4	113	23.8	
**M 2**							
no	390	46.3	168	45.7	222	46.8	0.393
yes	452	53.7	200	54.3	252	53.2	
**M 3**							
no	539	64.0	233	63.3	306	64.6	0.382
yes	303	36.0	135	36.7	168	35.4	

M1: scale ≥3 for receiving knowledge on one's own risk and options for surveillance factors

M2: scale ≥3 for reception of knowledge upon own risk, options for surveillance factors and the cause of cancer, M3: scale ≥3 for cause of one's own cancer, groups are not mutually exclusive, p-values refer to two-sided Chi2 tests. based on 842 non fast track persons (NFT)

Mothers want information about the reasons for their cancer more often than women without children (42.3 versus 29.2%). Women with lower education more often hope to learn reasons for their cancer diagnosis from the GC (56.1%) than those with higher education (36.4%). Additionally, women with a cancer diagnosis generally hope to receive information about their disease (65.2%) compared to those without a cancer diagnosis (10.9%). If a BRCA mutation had already been identified in the family, women are less likely to want to know whether the cancer is associated with genetics (59.1%) (as they already knew) compared to those without an unknown BRCA mutation status in the family (79.8%).

## Discussion

This prospective monocenter study looks at the sociodemographic and medical profiles of counselees attending the Heidelberg Center for HBOC, including referral practices and motivational factors prior to and after the AJE. Based on these descriptive data, suggestions to optimize genetic care supply have been gained. With a participating rate of 89%, the study population represents a standard collective for the HBOC, as all criteria had to be fulfilled prior to genetic consultation (GC), and includes counselees suffering from BC/OC and familial cases of HBOC.

The vast majority in this study were female (97.5%), which is also the case in other studies [17]. The gender distribution reflects the typical distribution of BC, as only 0.65% of cases are in males, although male BC patients face an elevated mutation risk and vice versa [18]. Our findings are also accompanied by the general under-use of genetic counselling by men with family HBOC, which has been previously described [19–21]. This could be due to interfamily communication and a lack of knowledge or understanding on the counselees’ part about autosomal dominant heritance because of the lack of surveillance program for men. It is known that the understanding of genetic topics varies wildly [22, 23], and a study has shown that men were often surprised about their personal risk and their probability to transmit a BRCA1/2 mutation to their children [21, 24],[25]. Beyond the understanding issues, the underrepresentation of men among HBOC counselees in Germany might additionally be due to the local care supply structure. Normally in Germany, the primary gynecologist provides a yearly check up, including breast care for women. This differs from other countries, where the general physician is in charge of such care standards; therefore men in Germany might miss out on genetic BC topics [26]. Particularly because screening adherence is known to be associated with primary care suppliers, this is congruent with our finding that the primary gynecologist is the main initiator for genetic testing. [27] We emphasize that the important role of primary care strategy deserves more attention, especially in respect to men with a HBOC history. Further, improvement strategies for knowledge-transfer between relatives are urgently needed as they have been described as highly defective and might miss people at risk [25]. We could show that in addition to primary gynecologists and hospitalists, family members play a major role in initiating genetic counseling referrals. Interestingly, women aged above 55 years referred themselves more often than younger women. This is in contrast to our expectations, but might be explained by the influence of family members or the starting age of population-based BC screening and the accompanying BC awareness among the elderly; it has been shown by others that cognitive awareness is associated with behavior [28].

Several other shortcomings have been discussed in the literature, such as disparities in service delivery for the appropriate identification of individuals with HBOC, including a lack of referral protocols, indicating a need for educational interventions for both providers and patients [29, 30]. Cragun et al. and others found that higher socioeconomic status, lower income and low physician referrals contribute to disparities in access to genetic services [31, 32], although we could not support the published finding in our cohort. Within Germany, any family member who meets the criteria of the HBOC for genetic consultation, including all family constellations with a mutation probability of >10% are eligible to undergo genetic consultation in Germany. Then, the consultation is covered by the general as well as private insurance companies and ensures an examination and participation in surveillance programs at one of the 17 HBOC centers.

The FT group is mostly referred by clinicians, as predicted. Referral is recommended in the tumor conference and then the genetic counseling initiated within the treatment concept for newly diagnosed BC in order to adapt treatment strategies. FT counselees had a lower waiting time, as expected. This did not change over time, and may have contributed to the increase in waiting time in the AFT group. This corresponds to the study by Knapke et al., which found good general availability of urgent cancer GC for exigent situations [33]. The waiting time in the NFT group increased significantly due to the AJE. As all subjects had to fulfil the HBOC criteria, it can be concluded that the correct target group (persons at high risk) has been reached by her actions as well as had a huge impact on public awareness of BRCA, spurring significant information seeking about BC genetic testing [34]. A persistent demand for GC even several months after indicates a long lasting effect, though awareness has not yet been associated with improved understanding [35–37]. A varying increase in referral practices, starting with a 2.5-fold increase, was noticed by others and supports our finding that the AJE encouraged family members with a known BRCA mutation to seek out the appropriate referral in order to gain for their families [16, 38]. We could show, in contrast to an investigation by Staudigl, that there was also an outreach among the male population [17]. The reduced number of people who cancelled their appointments also supports the theory that people felt this topic was personally relevant [16].

In general, the attendees are highly self-motivated and highly interested in most GC topics. Only a minority come exclusively on the recommendation of their physician or other care providers. The finding that women with a personal history of cancer are motivated to seek information on personal risk stratification emphasizes the need for a more personalized risk calculation, even for women who have already received a BC diagnosis, in order to address the varying contralateral BC/OC risks [3]. It is known that GC increases the accuracy of risk perception and decreases cancer-related worry, anxiety, and depression [39]. The desire to have an explanation for their personal cancer history, especially among those with a lower education as well as mothers, emphasizes the different understandings regarding cancer pathogenesis and genetic testing. These topics need to be addressed during consultation to meet those demands and outline the limits of current knowledge. Women with children, especially younger daughters, are found to have more concerns and therefore are more likely to attend a consultation [40]. Psychosomatic programs might be effective in improving quality of life and reducing distress and worries among these subgroups. Another important motivational factor to visit our HBOC centers is the interest in cancer surveillance programs for themselves and their family. Although recent data analysis suggests good adherence to the breast cancer surveillance program once started [6], we found that more than 30% of women didn’t participate at all (data not shown). Prospective studies are needed to further improve surveillance programs and optimize participation and adherence in respect to counselees’ expectations.

## Conclusions

In summary, we have found a higher number of participants, including more men, attending GC due to the AJE. In terms of the rising complexity of genetic testing, the analysis of patients’ expectations and initiators for GC suggests that there is an urgent need to develop medical educational programs for primary gynecologists and families at risk in order to motivate them to participate. (Social)media might be one attempt to provide well-structured information on basic genetics. However, these tools also need evaluation and approval by clinical trials.

## Supporting information

S1 File Questionnaire. (DOCX)

S1 Table Detailed information on initiators for genetic counselling. Referral patterns in the NFT and FT groups and the NFT subgroups “before AJ” and “after AJ”.
(DOCX)

S2 Table Expectations of FT counselees. Counselees quantified different expectations and motivational factors for visiting the Center for HBOC using a scale that ranged from 0 (motivation factor does not apply) to 4 (motivation factor applies completely).
(DOCX)
