# Supplementary material for: Familial breast cancer: Genetic counseling over time, including patients´ expectations and initiators considering the Angelina Jolie effect
Source: PLoS One. 2017 May 25;12(5):e0177893. doi: 10.1371/journal.pone.0177893 (PMC5444628; doi:10.1371/journal.pone.0177893)
Supplement: S1 File — (DOCX) [file pone.0177893.s001.docx]

S1 File:**Study Questionnaire**

**Care analysis for people at an increased risk for an familial cancer disposition**

Questionnaire for individuals from families with aggregation or early onset of breast and/or ovarian cancer seeking advice

1. When did you first learn about our genetic counselling unit on hereditary breast and ovarian cancer?

🞏 On approximately ______________ (please fill in the date)

🞏 Don’t know exactly, sometime before __________ (please fill in the approximate time frame)

🞏 Don’t know

1. When did you make your appointment with us?

🞏 On approximately ______________ (please fill in the date)

🞏 Don’t know

1. Is this the first appointment that you made?

🞏 Yes

🞏 No

1. Had you already had an appointment with us previously that you had to cancel?

🞏 Yes

🞏 No

1. Who long did you wait for your first appointment in the Genetics Clinic?

_________________ weeks

1. How far away is your place of residence from the Genetics Clinic?

_________________ km

1. Who suggested that you seek genetic counselling (multiple answers possible)?

🞏 a) Family doctor

🞏 b) Private practice gynecologist

🞏 c) Other medical specialist: which area? ________________________

🞏 d) Doctor at the University Gynecology Department in Heidelberg

🞏 e) Doctor from another hospital: which one? ___________________

🞏 f) Friend/relative

🞏 g) Me

🞏 h) Other: who? ____________________________________________

1. Only if you answered f, g, or h to question 7: How did you or the others learn about genetic counseling (multiple answers possible)?

🞏 Media (newspapers, television, radio)

🞏 Self-help group: Name?______________________________________

🞏 Word of mouth: Through whom?______________________________

🞏 Don’t know

🞏 Other: who?_______________________________________________

1. Where did you get the address/telephone number for the Heidelberg Genetics Outpatient Clinic from (multiple answers possible)?

🞏 a) Family doctor

🞏 b) Private practice gynecologist

🞏 c) Other medical specialist: which area? ________________________

🞏 d) Doctor at the University Gynecology Department in Heidelberg

🞏 e) Doctor from another hospital: which one? ___________________

🞏 f) Friend/relative

🞏 g) Self-help group: Name?___________________________________

🞏 h) Other: who? ____________________________________________

1. Which of the following statements apply to you in terms of your expectations of the consultation at the Genetics Outpatient Clinic? For each of the ten statements, please check the number that best describes your expectations. “0” means the statement does not apply to you at all, and “4” means that the statement applies to you fully.

| 1. I would like general information about hereditary breast and ovarian cancer | Does not apply at all  2  3  4  1  0  Applies fully |
| --- | --- |
| 1. I would like to know whether I have an increased cancer risk | Does not apply at all  2  3  4  1  0  Applies fully |
| 1. I would like to know whether my children and/or other relatives have in increased cancer risk | Does not apply at all  2  3  4  1  0  Applies fully |
| 1. I would like information about early cancer diagnosis options for myself | Does not apply at all  2  3  4  1  0  Applies fully |
| 1. I would like information about early cancer diagnosis options for my children and/or other relatives | Does not apply at all  2  3  4  1  0  Applies fully |
| 1. I would like to know why I have developed cancer | Does not apply at all  2  3  4  1  0  Applies fully |
| 1. I expect information on the inheritance of hereditary breast and ovarian cancer syndrome that could be of importance for my children | Does not apply at all  2  3  4  1  0  Applies fully |
| 1. I am not sure what my expectations are about the consultation at the Genetics Outpatient Clinic | Does not apply at all  2  3  4  1  0  Applies fully |
| 1. I am coming solely on the recommendation of my doctor/relative/other people | Does not apply at all  2  3  4  1  0  Applies fully |
| 1. None of the statements cover my expectations | Does not apply at all  2  3  4  1  0  Applies fully |

1. What is your gender?

🞏 Female

🞏 Male

1. Do you have children?

🞏 Yes

🞏 No

1. What is your marital status?

🞏 Single

🞏 Married

🞏 In a partnership

🞏 Divorced or separated

🞏 Widowed

1. What is your country of origin?

🞏 Germany

🞏 Other country

1. What type of town/city do you originate from?

🞏 Country-side/a village

🞏 A town

🞏 A large city

1. What is your highest attained grade-school certificate and professional certification (multiple answers possible):

🞏 No grade-school certificate

🞏 Completed 9 years of compulsory grade-school

🞏 Completed 10 years of compulsory grade-school

🞏 Completed 13 years of compulsory grade-school

🞏 Completed a vocational training program

🞏 Completed a degree at a university or college

1. What is your current occupational status?

🞏 A student in either grade-school, at university or in a training program

🞏 Employed

🞏 Freelance worker

🞏 Homemaker

🞏 Retired

🞏 Currently unemployed and not in any training

Thank you for your participation & help!
