# Supplementary material for: Familial breast cancer: Genetic counseling over time, including patients´ expectations and initiators considering the Angelina Jolie effect
Source: PLoS One. 2017 May 25;12(5):e0177893. doi: 10.1371/journal.pone.0177893 (PMC5444628; doi:10.1371/journal.pone.0177893)
Supplement: S1 Table — Referral patterns in the NFT and FT groups and the NFT subgroups “before AJ” and “after AJ”. (DOCX) [file pone.0177893.s002.docx]

**S1 Table: Detailed information on initiators for genetic counselling:** referral patterns in the NFT and FT groups and the NFT subgroups “before AJ” and “after AJ”

|  | | | | | | | | | | | | |  |  |  |
| --- | --- | --- | --- | --- | --- | --- | --- | --- | --- | --- | --- | --- | --- | --- | --- |
|  | |  |  |  |  |  |  |  |  |  |  |  |  |  |  |
| **Initiator** | **NFT** |  |  | **FT** | |  | **NFT before AJ** | | | **NFT after AJ** | | |  |  |  |
|  | N | % |  | N | % |  | N | % |  | N | % |  |  |  |  |
| **general physician** | 33 | 2.7 |  | 4 | 3.8 |  | 11 | 2.3 |  | 22 | 3.0 |  |  |  |  |
| **primary gynecologist** | 356 | 29.5 |  | 13 | 12.4 |  | 147 | 30.2 |  | 209 | 28.9 |  |  |  |  |
| **other specialist** | 61 | 5.0 |  | 1 | 1.0 |  | 29 | 6.0 |  | 32 | 4.4 |  |  |  |  |
| **University Heidelberg** | 144 | 11.9 |  | 44 | 41.9 |  | 63 | 13.0 |  | 81 | 11.2 |  |  |  |  |
| **other hospital** | 155 | 12.8 |  | 26 | 24.8 |  | 75 | 15,4 |  | 80 | 11.1 |  |  |  |  |
| **family/friends** | 217 | 18.0 |  | 6 | 5.7 |  | 82 | 16.9 |  | 135 | 18.7 |  |  |  |  |
| **oneself** | 165 | 13.7 |  | 5 | 4.8 |  | 57 | 11.7 |  | 108 | 15.0 |  |  |  |  |
| **other** | 77 | 6.4 |  | 6 | 5.7 |  | 22 | 4.5 |  | 55 | 7.6 |  |  |  |  |
| **total** | 1208 |  |  | 105 |  |  | 486 |  |  | 722 |  |  |  |  |  |

based on 842 persons without fast track (NFT) and 75 fast track (FT) patients, before and after AJ, refers to August 31st 2013, multiple entries possible
