# Supplementary material for: Familial breast cancer: Genetic counseling over time, including patients´ expectations and initiators considering the Angelina Jolie effect
Source: PLoS One. 2017 May 25;12(5):e0177893. doi: 10.1371/journal.pone.0177893 (PMC5444628; doi:10.1371/journal.pone.0177893)
Supplement: S2 Table — Counselees quantified different expectations and motivational factors for visiting the Center for HBOC using a scale that ranged from 0 (motivation factor does not apply) to 4 (motivation factor applies completely). (DOCX) [file pone.0177893.s003.docx]

**S2 Table: Expectations of FT counselees:** counselees quantified different expectations and motivational factors for visiting the Center for HBOC using a scale that ranged from 0 (motivation factor does not apply) to 4 (motivation factor applies completely).

|  | **0** |  |  | **1** |  |  | **2** |  |  | **3** |  |  | **4** |  |  | **missing** | |
| --- | --- | --- | --- | --- | --- | --- | --- | --- | --- | --- | --- | --- | --- | --- | --- | --- | --- |
|  | **N** | **%** |  | **N** | **%** |  | **N** | **%** |  | **N** | **%** |  | **N** | **%** |  | **N** | **%** |
| **General information (HBOC)** | 7 | 9.3 |  | 4 | 5.3 |  | 8 | 10.7 |  | 7 | 9.3 |  | 47 | 62.7 |  | 2 | 2.7 |
| **Own cancer risk** | 7 | 9.3 |  | 0 | 0.0 |  | 2 | 2.7 |  | 1 | 1.3 |  | 63 | 84.0 |  | 2 | 2.7 |
| **Cancer risk for relatives** | 4 | 5.3 |  | 2 | 2.7 |  | 5 | 6.7 |  | 3 | 4.0 |  | 59 | 78.7 |  | 2 | 2.7 |
| **Own surveillance** | 13 | 17.3 |  | 3 | 4.0 |  | 9 | 12.0 |  | 8 | 10.7 |  | 39 | 52.0 |  | 3 | 4.0 |
| **Surveillance for relatives** | 8 | 10.7 |  | 3 | 4.0 |  | 7 | 9.3 |  | 13 | 17.3 |  | 42 | 56.0 |  | 2 | 2.7 |
| **Cancer aetiology** | 7 | 9.3 |  | 7 | 9.3 |  | 10 | 13.3 |  | 7 | 9.3 |  | 43 | 57.3 |  | 1 | 1.3 |
| **Family planning** | 34 | 45.3 |  | 1 | 1.3 |  | 6 | 8.0 |  | 7 | 9.3 |  | 24 | 32.0 |  | 3 | 4.0 |
| **No personal expectations** | 42 | 56.0 |  | 11 | 14.7 |  | 13 | 17.3 |  | 4 | 5.3 |  | 3 | 4.0 |  | 2 | 2.7 |
| **Visit on recommendation** | 40 | 53.3 |  | 11 | 14.7 |  | 13 | 17.3 |  | 4 | 5.3 |  | 5 | 6.7 |  | 2 | 2.7 |
| **Other expectations than above** | 66 | 88.0 |  | 1 | 1.3 |  | 2 | 2.7 |  | 0 | 0.0 |  | 1 | 1.3 |  | 5 | 6.7 |

|  |
| --- |

scale 0 - 4 from "completely no" to "completely yes", row percentages; based on 75 fast track (FT) patients
